# Experimental annotation of the human pathogen Histoplasma capsulatum transcribed regions using high-resolution tiling arrays

**DOI:** 10.1186/1471-2180-11-216

**Published:** 2011-09-29

**Authors:** Mark Voorhies, Catherine K Foo, Anita Sil

**Affiliations:** 1 Department of Microbiology & Immunology, University of California San Francisco, San Francisco, California, 94143, USA; 2 Department of Biochemistry and Biophysics, University of California San Francisco, San Francisco, California, 94143, USA; 3 Howard Hughes Medical Institute, University of California San Francisco, San Francisco, California, 94143, USA

## Abstract

**Background:**

The fungal pathogen *Histoplasma capsulatum *is thought to be the most common cause of fungal respiratory infections in immunocompetent humans, yet little is known about its biology. Here we provide the first genome-wide studies to experimentally validate its genome annotation. A functional interrogation of the *Histoplasma *genome provides critical support for continued investigation into the biology and pathogenesis of *H. capsulatum *and related fungi.

**Results:**

We employed a three-pronged approach to provide a functional annotation for the *H. capsulatum *G217B strain. First, we probed high-density tiling arrays with labeled cDNAs from cells grown under diverse conditions. These data defined 6,172 transcriptionally active regions (TARs), providing validation of 6,008 gene predictions. Interestingly, 22% of these predictions showed evidence of anti-sense transcription. Additionally, we detected transcription of 264 novel genes not present in the original gene predictions. To further enrich our analysis, we incorporated expression data from whole-genome oligonucleotide microarrays. These expression data included profiling under growth conditions that were not represented in the tiling experiment, and validated an additional 2,249 gene predictions. Finally, we compared the G217B gene predictions to other available fungal genomes, and observed that an additional 254 gene predictions had an ortholog in a different fungal species, suggesting that they represent genuine coding sequences.

**Conclusions:**

These analyses yielded a high confidence set of validated gene predictions for *H. capsulatum*. The transcript sets resulting from this study are a valuable resource for further experimental characterization of this ubiquitous fungal pathogen. The data is available for interactive exploration at http://histo.ucsf.edu.

## Background

**Figure 1:**
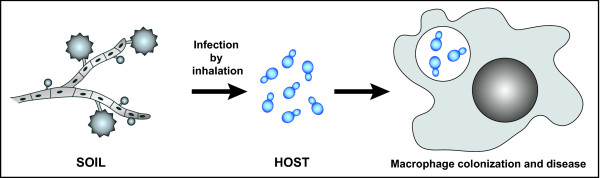
**Histoplasma capsulatum is a dimorphic fungal pathogen**. *Histoplasma capsulatum *grows as a saprophytic mold in the soil (left) but, upon inhalation by a mammalian host, converts to a pathogenic yeast form (center) capable of intracellular growth within host macrophages (right). Both small and large vegetative spores (micro and macroconidia, respectively) are depicted in the mold form. Within the macrophage, yeast cells are shown within a membrane-bound phagosome, and the macrophage nucleus is also depicted.

*Histoplasma capsulatum *is a dimorphic fungal pathogen that is thought to infect up to 500,000 individuals per year in the U.S[1]. Notably, *H. capsulatum *is a primary pathogen that causes significant morbidity in immunocompetent hosts[2]. Normally found in a filamentous mycelial form in the soil of endemic regions, *H. capsulatum *converts to the pathogenic yeast form in the lungs of the host after inhalation of infectious particles (Figure 1). In the laboratory, temperature is a sufficient signal to specify growth in either the mycelial form (at room temperature) or growth in the yeast form, which can be achieved by incubating cells at 37°C. Once introduced into the host, *H. capsulatum *colonizes host immune cells. Understanding both how *H. capsulatum *switches its growth program in response to temperature and how this pathogen subverts the innate immune system are major areas of inquiry.

The elucidation of *H. capsulatum *pathogenesis and biology has been greatly aided by the genome sequencing of *H. capsulatum *strains G217B and G186AR at the Genome Sequencing Center (GSC) at Washington University in St. Louis and strains G186AR, WU24, H88, and H143 at the BROAD Institute. These sequenced genomes open up a wealth of possibilities for the *H. capsulatum *community, enabling or abetting tools such as expression arrays, insertional mutagenesis, and bioinformatic analysis. However, these approaches are limited by the gene annotations associated with the genome assemblies. This limitation is pronounced in *H. capsulatum *given this eukaryote's sparse gene structure and a limited set of known transcripts with which to train gene prediction algorithms. Accordingly, although the GSC used a variety of tools to generate a set of predicted genes for G217B and G186AR http://genome.wustl.edu/genomes/view/histoplasma_capsulatum/, these predictions are based on limited experimental data.

In other systems where the gene finding problem has presented itself, whole genome tiling has proven a reliable technique for direct observation of the transcriptome[3-6]. To this end, we generated a set of tiling microarrays spanning the non-repetitive regions of the G217B genome and hybridized these arrays with a pool of cDNA derived from yeast-form *Histoplasma *growing under a diverse set of conditions. The resultant data give an unbiased measure of expression level as a function of genome position, and thus identify the locations and boundaries of expressed genes. The results of this study are available, along with tools for interactive exploration of the data, at http://histo.ucsf.edu.

## Results and Discussion

### Whole-genome tiling array expression profiling

**Figure 2:**
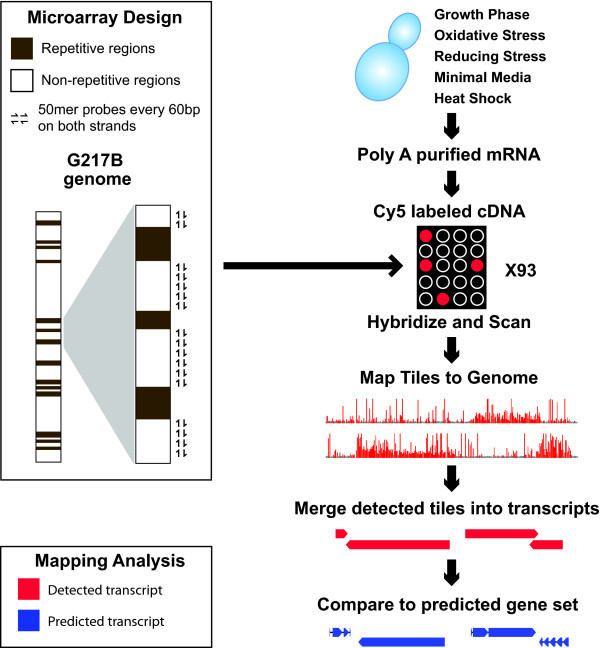
**Characterization of the Histoplasma capsulatum transcriptome by whole genome tiling arrays**. mRNA from diverse yeast conditions (top right) was used to prepare labeled cDNA which was then hybridized to 93 Combimatrix tiling arrays with 50 mer probes spanning the non-repeat G217B genome (left). The resulting signal was kernel-smoothed to yield a detected transcript set, which was compared to the predicted gene set (bottom).

To survey the transcriptome of G217B, we designed a set of 93 unique tiling microarrays (Figure 2). The G217B genome contains a large number of repeat regions, including the MAGGY retrotransposon[7], which were excluded from the tiling microarray probes. Both strands of the remaining sequence were tiled with 50 mer probes at an average frequency of one probe every 60 base pairs (Figure 2). These arrays were hybridized with a pool of fluorescently labeled cDNA generated from cells grown under a variety of conditions. Because technical limitations did not allow us to isolate sufficient poly-adenylated-RNA from filamentous cells (which represent the soil form of this organism and must be grown under biosafety level three conditions due to the production of aerosolizable infectious spores), we focused on the pathogenic yeast form. G217B yeast cells were subjected to numerous growth conditions (see Materials and Methods) which had previously been observed to elicit potent transcriptional responses[8,9]. Tiles that passed an empirically determined detection threshold were merged into TARs, as described in the Materials and Methods.

### Detection of predicted genes

**Figure 3:**
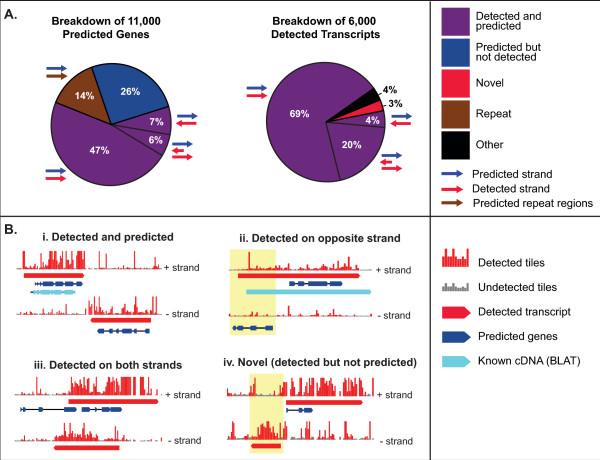
**Detected transcripts correspond to predicted genes**. A) Coverage of predicted genes by detected transcripts (left) and of detected transcripts by predicted genes (right). Arrows next to sectors of the pie charts indicate the relative orientation of predicted genes (blue), detected transcripts (red), and repeat regions (brown). B) Representative cases for coincidence of detected transcripts with predicted genes. Features: detected (red) and undetected (gray) tiling signal (vertical bars), detected transcripts (red), predicted genes (blue), and experimentally mapped cDNAs (cyan). Areas of interest in ii and iv are highlighted with a yellow rectangle.

The GSC predicted that the G217B genome contains 11,221 genes, but 1,611 of these gene predictions contain repeat sequence, including the MAGGY transposon, and were excluded from further analysis. Of the remaining 9,610 predictions, 6,008 were detected in our tiling microarrays (Figure 3a). 60% of the gene predictions have some correspondence to the detected TARs: 47% of the predictions were cleanly detected only on the predicted strand (represented in Figure 3b i), 7% were detected only on the antisense strand (Figure 3b ii), and 6% had tiling and/or prediction support for transcription on both strands (Figure 3b iii), leaving 26% of the predicted set unsupported by our tiling data (Figure 3a). Detection on both strands is consistent with the presence of sense and/or antisense transcripts in one or more of the growth conditions profiled by this experiment. It has been shown that the DNA-dependent DNA polymerase activity of reverse transcriptase can generate false positive opposite strand signal in tiling experiments; *e.g*., two thirds of putative antisense transcripts in a *Saccharomyces cerevisiae *tiling experiment were not detected in the presence of actinomycin D[10]. Therefore, the number of sense/antisense pairs observed in our experiment is likely to be an overestimate.

Detection on only the antisense strand may correspond to incorrect predictions coinciding with *bona fide *transcripts on the opposite strand (*e.g*., Figure 3b iii, in which there is a spurious prediction antisense to the known 5' UTR of FDH1[9]) or to true genes that are repressed by an antisense transcript in our pooled yeast sample. Due to this ambiguity, genes in this category were not considered "detected". An additional 264 novel transcripts, which were not present in the predicted set, were also detected (Figure 3b iv), as described below. As part of the web database associated with this study, the detected transcript set can be viewed in the context of the raw tiling signal and predicted gene set (as in Figure 3), allowing human estimation of transcript set accuracy on a case by case basis.

### Features of transcribed regions in the *H. capsulatum *genome

**Figure 4:**
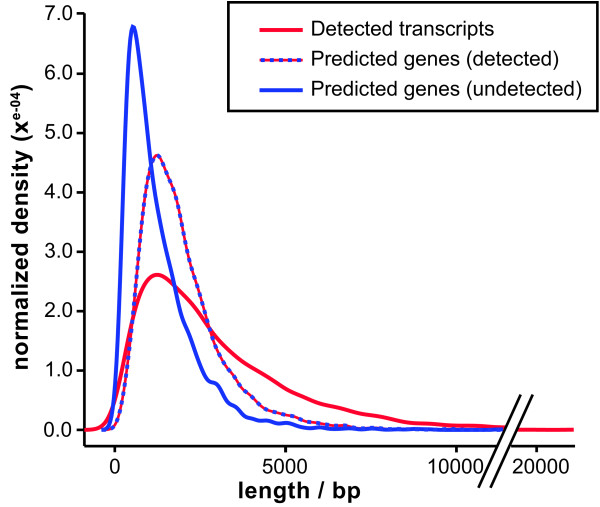
**Length of predicted genes correlates with detection**. Normalized length distributions for detected TARs (red) and predicted genes that were undetected by any method (blue) or detected by at least one method (dashed red and blue).

As is common for tiling data, the boundaries of TARs did not correspond precisely with the boundaries of the predicted genes. There were two common instances of this pattern. First, in many cases, additional transcription was detected 5' and 3' of the predicted gene (Figure 3b). This was most likely due to untranslated (UTR) sequences which are missed by the gene model and resulted in a longer length distribution for the TARs compared to the predicted genes (Figure 4). Second, it was not uncommon for a single long transcript to span multiple predictions. In some cases, this was due to the sequence encoding a single TAR being incorrectly predicted to contain multiple genes. In others, this was due to multiple genes being incorrectly detected as a single transcript, either due to spurious or pathological background signal or due to intergenic regions too small to be distinguished from introns. In the case of the *Saccharomyces cerevisiae *genome, multi-gene detected transcripts could be segmented based on sharp transitions in the intensity of the tiling signal[11]. Such analysis would be difficult in the present study, primarily because the tiling sample is a pool of cDNAs corresponding to multiple transcriptional states of the *H. capsulatum *yeast phase, each of which may contain transcript isoforms that differ by splicing and transcriptional start site (we have documented such variability for several phase specific transcripts in *H. capsulatum*[9]). Ultimately, we attempted to minimize this limitation of the tiling array method by selecting transcript detection parameters that distinguish the mostly small introns from the mostly large intergenic regions.

The majority of TARs that did not overlap with gene predictions corresponded to unpredicted UTR sequences. For example, 29% of non-overlapping TAR sequence can be interpreted as 5'UTR (immediately upstream of and contiguous with a gene prediction), and 35% as 3'UTR (immediate downstream of and contiguous with a gene prediction). Additionally, 33% of non-overlapping TARs corresponded to the intervening sequence between two predictions (*i.e*., intergenic sequence incorrectly detected as transcribed due to the resolution limits of the tiling strategy, or long transcripts incorrectly predicted as multiple genes).

### Tiling arrays revealed 264 novel genes

One advantage of a tiling strategy is that it can uncover novel TARs that do not correspond to the predicted genes. Our tiling analysis detected 264 such loci that were not represented in the GSC predicted gene set for G217B (*e.g*., Figure 3b iv). TARs were designated as "novel" if (1) they had no overlap with gene predictions on either strand, (2) they did not fall into the "5'UTR", "3'UTR", or "intervening" classifications described above (*i.e*., the flanking 5' and 3' base did not coincide with a gene prediction), and (3) they had no overlap with repeat regions.

**Figure 5:**
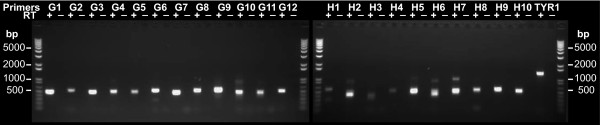
**Novel transcripts are validated by RT-PCR**. RT-PCR products for primer pairs targeting *TYR1 *(positive control) and 22 novel transcripts detected on the whole genome tiling arrays. A standard DNA ladder flanks each gel. "RT" indicates whether reverse transcriptase was added to the cDNA synthesis reaction.

94 TARs that did not coincide with the predicted gene set were chosen for experimental validation by RT-PCR. 79 of these TARs were detected in a first-pass analysis with a single primer pair, giving a validation rate of 84%. A representative sampling of RT-PCR results is shown in Figure 5.

To determine whether the novel loci correspond to conserved sequences in other genomes and, if so, if these homologous loci have been independently annotated as transcribed (*i.e*., if they are merely unannotated in G217B), we searched for conserved sequences in other dimorphic fungal pathogens within the order Onygenales (4 strains of *H. capsulatum*, 2 strains of *Blastomyces dermatitidis*, 3 strains of *Paracoccidioides brasiliensis*, and the reference strain of *Coccidioides immitis*).

**Figure 6:**
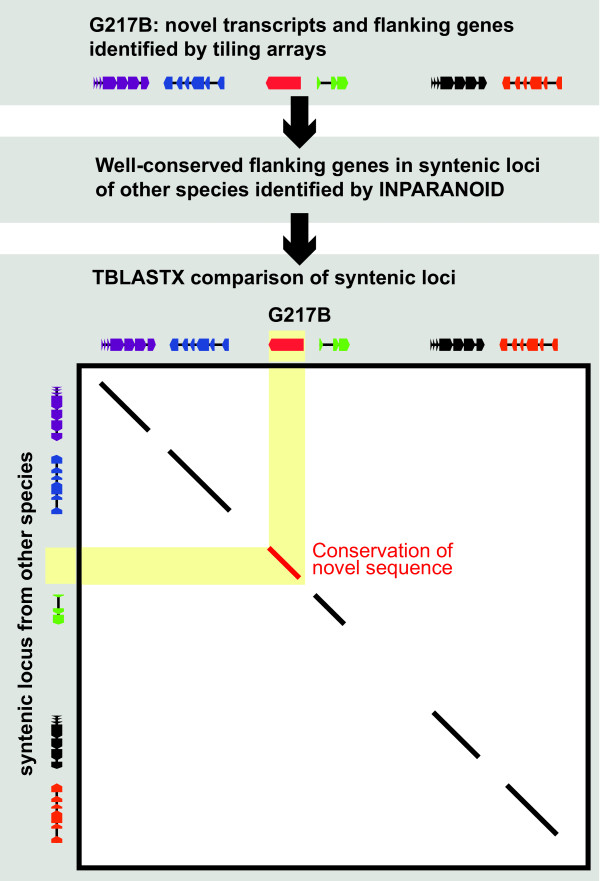
**Syntenic loci were mapped using an INPARANOID-based strategy**. As described in the results section, syntenic loci were defined as non-G217B contigs containing INPARANOID-based orthologs (y-axis) of genes within 20 kb of novel TARs (x-axis). TBLASTX was used for comparison of the syntenic loci at the sequence level (main diagonal plot). The red transcript represents the novel TAR. Each of the other colors represents an ortholog pair in the two species.

A BLASTX search of the isolated novel sequences against the predicted protein sets yielded a number of hits in other genomes (173 of the isolated novel sequences had hits in at least one non-G217B *H. capsulatum *gene set, and 63 of these had hits in at least one non-*H. capsulatum *gene set). To increase the sensitivity of this search, we performed an INPARANOID-based[12] mapping of syntenic loci that flanked each novel locus (Figure 6). Genes in 20 kb windows on either side of the novel TAR could be independently mapped to orthologs on a common contig in at least 8 other genomes for 217 of the isolated novel sequences. Of the 173 isolated novel sequences with BLASTX hits, 156 could be mapped to syntenic loci, and, for 150 of these, the BLASTX hit coincided with the mapped locus. A TBLASTX (translated nucleotide vs. translated nucleotide) comparison of the isolated novel sequence to the mapped locus yielded a significant alignment (*e *≤ 10^-6^) for at least 4 *H. capsulatum *strains in 210 cases, for both *B. dermatitidis *strains in 80 cases, for at least two *P. brasiliensis *strains in 31 cases, and for the reference *C. immitis *strain in 7 cases. In general, the TBLASTX results were consistent with evolutionary distance from G217B (*e.g*. sequences conserved between *H. capsulatum *and *B. dermatitidis *were also conserved among *H. capsulatum *strains).

Taken together, these results suggest that: 1) the isolated novel sequences are conserved at the sequence level, and, therefore, likely to be transcribed, relative to the other *H. capsulatum *strains in most cases, and relative to *B. dermatitidis *for about half of the cases; 2) transcripts with deeply conserved sequence across the Onygenales also tend to be predicted as genes in most of these fungi; and 3) for about half of the isolated novel sequences, a corresponding gene prediction exists in another genome, highlighting differences in the prediction pipelines, while the other half represent truly novel discoveries of this tiling experiment.

### Using standard expression profiling and sequence homology to enrich gene validation

**Figure 7:**
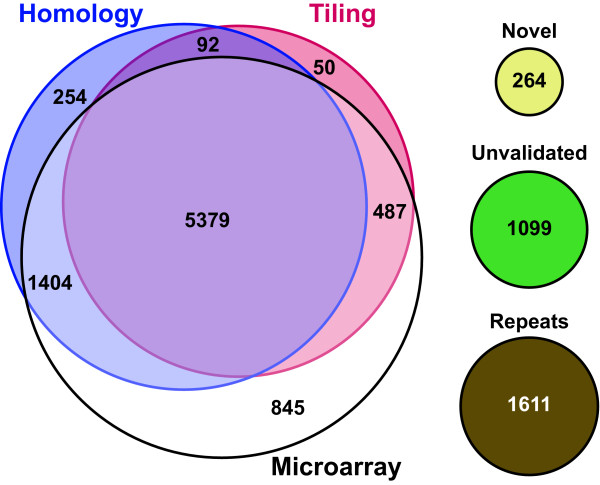
**A majority of predicted genes are validated by multiple methods**. Summary of genes validated by tiling (red), homology (blue), or expression (white). The circles on the right indicate special, disjoint classes: novel, tiling-detected transcripts with no corresponding gene prediction (yellow); predicted genes not validated by any method (green); and predicted genes with significant overlap to repeat regions (excluded from the analysis) (brown).

To complement our tiling arrays, we took advantage of our archive of expression data compiled across several distinct growth conditions, including iron limitation, and all three morphologies (yeast, mycelia, and conidia). We surveyed whether gene predictions were detected in these expression profiling experiments, which employed whole-genome oligonucleotide microarrays where each prediction was represented by one or two gene-optimized 70 mer probes. Additionally, we used INPARANOID[12] to determine if gene predictions had homologs in other fungi. This validation by inferred homology to genes in other fungi relied on sequence conservation independent of expression pattern. The validation criteria for each strategy are given in the methods section and the results are summarized in Figure 7 (detailed per-gene results are available as Additional file 1, Table S1 and may be browsed interactively at http://histo.ucsf.edu). By these criteria, 8,115 non-repeat predicted proteins were validated by gene expression and 7,129 were validated by sequence homology.

Genes that were validated by tiling, gene expression, and sequence homology represented the largest category of predictions (5,379 genes) and accounted for 56% of the non-repeat predicted gene set. The next largest category was 1,404 genes validated by gene-expression and sequence conservation but not by the tiling experiment (15% of the non-repeat predicted gene set), followed by 845 genes (9%) validated only by expression array, and 487 genes (5%) validated by expression and tiling but not sequence conservation. 1,099 gene predictions (11%) were unvalidated by any of the three methods.

In the following discussion, predicted genes are referred to by their common names. Additional file 2, Table S2 gives the corresponding systematic names.

#### Genes that were missed by tiling array showed enriched expression in the mycelial form

As expected, gene predictions that were not detected by tiling tended to show reduced expression in the yeast phase and enhanced expression in the mycelial form. Examples include *TYR1 *and *ABC4*, both previously identified as highly enriched in the mycelial phase [9]; *VELC*, a mycelial-enriched paralog of the morphological regulators *RYP2 *and *RYP3 *[13]; and the ortholog of BDBG_03463, which is paralogous to the *B. dermatitidis *gene *BYS1 *(*BYS1 *itself has no ortholog in *H. capsulatum*)[14,15].

Other notable categories of genes not detected by tiling include genes in heavily repeat-masked regions of the genome (where the tiling density is, therefore, too low to analyze) and genes with weak expression that did not give significant signal over background on tiling arrays.

#### Genes that were not detected by homology represented short or interrupted predictions

For genes not detected by homology, there were two related trends: (1) the predicted lengths were short, on the order of those genes not detected by any method (Figure 4); and/or (2) a single TAR was inappropriately split into multiple predicted genes. For example, the copper-repressed gene *ELI1*, which is known to be expressed as a single mRNA[16], is split into two predictions. Both predictions are detected by expression and tiling, but only the 3' prediction, which contains the coding sequence, is detected by homology, whereas the 5' prediction, which likely contains 5'UTR, is not. Short predictions are difficult to detect as homologs for two reasons: short runs of sequence similarity are likely to occur by chance, resulting in lower BLASTP p-values for hits to these predictions; and INPARANOID requires 50% reciprocal coverage between orthologs, resulting in rejection of genes where the predicted length is significantly smaller than that of the corresponding homologs. The same issues arise for split predictions, with the additional restriction that INPARANOID will make an ortholog assignment for only one member of a split pair, automatically resulting in rejection of the other member.

In all of these cases, the discrepancy between the experimental and sequence based results is a useful indication that the predicted gene model should be revised. In many cases, comparison of the transcript detected by tiling array to the results of less stringent sequence searches (*e.g*., BLASTX of the transcribed genomic sequence) is a useful starting point for such revision.

Genes not detected by homology also tend to show enriched expression in conidia, the vegetative spores generated by *H. capsulatum *filaments. *H. capsulatum *conidia, or their counterparts in any closely related fungi, have not been extensively studied; thus, the homologs of these genes may be unpredicted or entirely absent in the comparison genomes.

#### Genes that were validated only by homology have restricted expression profiles

The category of genes with orthologs in other fungi but no direct observation in our experimental data was relatively small (254 predictions representing 3% of the non-repeat gene set) and is predicted to contain genes that are expressed only under very restricted conditions that were not sampled in our expression data. Consistent with this hypothesis, we find *STE3*, the a-factor receptor whose expression has been observed only in mutants of G217B[17]; the ortholog of *N. crassa RID*, which is required for the RIP process and therefore expected to be expressed only during meiosis[18]; and the ortholog of *T. reesei AXE2*, a hemicellulolytic enzyme whose expression is dependent on carbon source[19].

#### Empirical redesign of microarray probes

Our tiling arrays and homology predictions can be used to inform future design of microarray probes. Because the expression experiments draw from a more diverse set of samples than the tiling experiments, detection of a predicted gene by homology and tiling but not by expression suggested a platform-specific defect in the 70 mer probe designed to detect that gene on our whole-genome oligonucleotide arrays (rather than a failure of the expression experiments to sample the appropriate condition). Our analyses support this hypothesis. In particular, the 70-mer probes for genes that failed to be detected by expression array tend to lie outside of the transcribed locus detected by tiling (*e.g*., the nitrositive-stress induced transcript *COX12*[8]), or span a predicted intron not supported by the tiling data (*i.e*., due to incorrect gene prediction, the 70 mer probe targets a discontiguous sequence in the true transcript). We are currently augmenting the expression array platform with new 70 mers for these genes, based on the coincidence of tiling transcripts with predicted exons.

#### Genes that failed to be validated by any method

We were unable to validate 1,099 predictions, or 11% of the non-redundant genes, by any method. This group primarily corresponds to wholly undetected predictions but may also include a small number of correct predictions for which the 5' end is undetected due to the 3' bias of the tiling experiment.

The unvalidated genes are significantly shorter than the detected genes (Figure 4). This observation could be due to false negatives in the tiling data (short transcripts are more difficult to detect because they are difficult to distinguish from background noise) or false gene predictions (there is an increased likelihood of short sequences fitting a gene model by chance). We note that genes validated only by expression (our only validation method that is independent of transcript length) are significantly shorter than genes validated by all methods but significantly longer than the unvalidated genes, lending weight to both explanations.

## Conclusions

We probed the transcriptome of *H. capsulatum *with a large set of tiling arrays, and combined the results with gene-targeted expression profiling and sequence homology, yielding a high confidence set of validated gene predictions for G217B with 7,362 gene predictions being validated by at least two of the three methods. In addition, the unbiased approach of the tiling arrays allowed us to detect 264 novel transcripts that are now being incorporated into our oligo expression arrays, directly extending the sensitivity of that platform. Additionally, the results of this study are available at http://histo.ucsf.edu in an interactive format intended to facilitate expression, insertional mutagenesis, and bioinformatics based studies. Thus, the transcript sets resulting from this study represent an enhancement of the previously available *H. capsulatum *gene set and a starting point for the experimental and theoretical characterization of the molecular biology of this important intracellular pathogen.

## Methods

### RNA Extraction and cDNA synthesis

To generate a diverse RNA sample for the tiling experiment, we prepared RNA from yeast-form *Histoplasma capsulatum *strain G217B (ATCC 26032; a kind gift of William Goldman, Washington University, St. Louis, MO) under a variety of conditions (including early, middle, and late logarithmic growth, stationary phase, heat shock (42°C for 30 min), oxidative stress (1 mM menadione for 80 min), sulfhydryl reducing stress (10 mM DTT for 2 hours), and a range of media (HMM[20], 3M[20], YPD[21], and SD complete[21]). Total RNA and polyA RNA were prepared as previously described[8,9]. Cy5-labeled cDNA was prepared from individual RNA samples as previously described[8], and an equal mass of cDNA was pooled from each sample and hybridized to individual tiling arrays as described below.

### Whole Genome Tiling Array Design

The whole genome tiling arrays were designed based on the GSC *Histoplasma capsulatum *strain G217B genome assembly as of 11/30/2004. Degenerate sequence and transposable elements were removed from the assembly using RepeatMasker[22] with default parameters and the repeat families determined by the GSC. The remaining sequence was tiled with 50 mer probes at an average frequency of one probe every 60 base pairs. Probe spacing was adjusted to minimize variation in melting temperature, and a subset of probes were truncated to optimize synthesis, in collaboration with CombiMatrix. The number of arrays used to tile a given contig was minimized, and the location of tiling probes was randomized within a given array.

In addition, each array contained a common set of control probes, *viz*.: quality control (QC) and negative control (NC) probes designed by CombiMatrix (Mukilteo, WA); positive control probes tiling the genomic loci and non-genic flanking sequence of *TEF1*(P40911)[23], *TYR1*[9], and *CBP1*(AF006209)[24]; and probes specific to a spike-in control sequence. The QC, NC, and spike-in probes were not considered in the analysis.

### Hybridization of tiling arrays

Fluorescently labeled cDNA was hybridized to CombiMatrix arrays as previously described[8]. In addition to the Cy5-labeled sample described above, a common Cy3-labeled sample was used as a counterpoint reference on each array.

Images of the hybridized arrays were acquired with a GenePix 4000B scanner (Axon Instruments) controlled by the GenePix 4.0 program (Molecular Devices). Each array was scanned three times using the following PMT settings for the 635 nm laser: 400, 450, 540. Images were gridded with GenePix 4.0 and the median foreground intensity for each feature was used as the input for subsequent analysis. Based on the negative control probes, signal/noise was constant for the three scans, so all subsequent analysis was carried out using the lowest PMT scan.

### Probe detection on tiling arrays

Background intensity was estimated based on the median intensities of a control set of known antisense and intergenic regions, a method similar to the use of median intensities of known introns in the analysis of rice tiling data[6]. Specifically, the background intensity was estimated as the median intensity of the positive control probes corresponding to the intergenic (untranscribed) regions flanking *CBP1 *and *TYR1 *and the antisense (untranscribed) probes for *CBP1*, *TYR1*, and *TEF1*. A tiling probe was considered detected if it had intensity greater than the background intensity estimated for the corresponding array. 58% of the tiling probes were considered detected by this method.

### Transcript detection on tiling arrays

In *H. capsulatum*, introns are small enough to make detection of complete transcripts feasible (in contrast to, *e.g*., *Homo sapiens*) but are large and irregular enough to make such detection non-trivial (in contrast to, *e.g*., *Escherichia coli *or *Saccharomyces cerevisiae*). For this study, we traded resolution for improved signal to noise and defined transcripts as genomic loci ≥ 200 bp for which the normalized density of detected probes was greater than 65% of the normalized density of all probes. Smoothed densities were calculated with the density function in R[25] using a bandwidth of 500 bp, and transcripts were truncated such that transcript ends coincided with detected tiles.

In order to avoid regions of the tiling path that were rendered sparse due to repeat masking, transcript detection was restricted to regions spanning at least 10 kb of genome sequence with a minimum tiling density of 1 probe per 250 bp (1/5*^th ^*of the target tiling density).

6,172 transcripts were detected. The length distribution (in terms of genomic locus) for detected and predicted transcripts is shown in Figure 4. Known transcripts showed a mild 3' bias, meaning that signal intensity was enriched at the 3' end of the gene, as expected given the method of sample preparation.

The genomic coordinates of the detected transcripts are given in Additional file 3, Table S3, and the probe intensities are given in Additional files 4 and 5, Data S4 and Data S5.

### RT-PCR

94 novel TARs were examined by RT-PCR. Primers were designed using the Primer3[26] program (with the Primer3plus[27] default parameters) to design up to 5 primer pairs (giving 400-500 bp products) for each transcript. The designed primer pairs were then screened for redundant products using the re-PCR[28] program with the first non-redundant pair being chosen for each target (targets with 5 redundant pairs were rejected).

PolyA RNA corresponding to the cDNA used for tiling arrays was subjected to RT-PCR analysis, with the exception that RNA from early log-phase cells was not included due to limited material. The pooled RNA was DNAse treated and reverse transcribed with AffinityScript (Stratagene). PCR reactions were carried out using AmpliTaq polymerase (Applied Biosystems) for 35 cycles of [94°C 15" → 56°C 15" → 72°C 4'].

Reaction products were visualized on a 1% agarose gel and were considered detected if they occurred at the length predicted by the re-PCR program with no corresponding band in the "no RT" control.

The sequences of the full set of novel TARs are given in Additional file 6, Data S6.

### Gene validation

For the purpose of validation, the length of a predicted gene was taken as its full genomic locus (including introns and exons).

RECON[29]-identified repeat-families from the GSC (including the MAGGY transposon[7]) were mapped to the genome with REPEATMASKER[22] using default settings and excluding simple sequence repeats. Predicted genes with greater than 20% of their length covered by REPEATMASKER-annotated repeat sequence were classified as repeats and removed from further analysis.

Non-repeat genes with greater than 50% of their length covered by detected TARs were classified as validated by tiling.

The following two-channel G217B whole-genome oligonucleotide microarray data sets were used for validation by expression profiling: wild type and *ryp1 *mutant 37°C and RT samples hybridized against a pooled reference (9 arrays[30]), direct hybridizations of yeast, mycelial, and conidial samples (6 arrays, Inglis *et al*, unpublished), iron depletion time courses hybridized against a pooled reference (8 arrays[31] plus 10 arrays, Hwang *et al*, unpublished). In keeping with our standard analysis pipeline for this platform, probes were considered detected if they were not manually flagged as bad and the sum of background-subtracted median intensities for the two channels was greater than 500. Non-repeat predicted genes were classified as validated by expression array if they mapped to at least one detected probe in at least 3 of the 33 arrays.

Annotated gene sets from the following genomes were used for validation by homology to other fungi: *Blastomyces dermatitidis *er-3 and slh14081; *Paracoccidioides brasiliensis *pb01, pb03, and pb18; *Coccidioides immitis *rs; *Aspergillus nidulans*; *Aspergillus fumigatus *(TIGR); *Aspergillus oryzae *(DOGAN); *Neurospora crassa*; *Magnaporthe oryzae *(formerly *Magnaporthe grisea*); *Fusarium graminearum*; *Candida albicans *(CGD, orfs19 gene set); *Saccharomyces cerevisiae *(SGD); *Cryptococcus neoformans *H99; and *Ustilago maydis*. Except where noted, all gene sets were obtained from the BROAD Institute. Pairwise ortholog/in-paralog mapping to G217B was performed by running INPARANOID[12] with default parameters and no outgroup for each genome. Predicted genes were classified as validated by homology if they were a member of an orthogroup (direct ortholog to a gene in the target genome or in-paralog of a G217B gene with a direct ortholog in the target genome) for at least 3 of the 16 target genomes.

### Accession codes

Microarray data have been submitted to the NCBI Gene Expression Omnibus (GEO) under accession number [GEO:GSE31155].

Nucleotide sequence data for the reported novel TARs are available in the Third Party Annotation Section of the DDBJ/EMBL/GenBank databases under the accession numbers TPA: BK008128-BK008391.

## Authors' contributions

CKF designed the whole genome tiling arrays, prepared the RNA samples and performed the microarray experiments. MV and AS analyzed the data and performed the RT-PCR experiments. MV, CKF, and AS prepared the manuscript.

All authors read and approved the final manuscript.

## Supplementary Material

Additional file 1 **Table S1**. CSV formatted table of gene validation results, corresponding to the classification n Figure 7. Columns: *gene *- GSC predicted gene name, *NAm1ortholog *- BROAD gene name for the INPARANOID identified ortholog in *H. capsulatum *WU24, *repeat*, *wgtaValid*, *exprValid*, and *orthoValid *- 1 if a gene was classified as repeat or validations by tiling, expression, or homology respectively; 0 otherwise. Sequences (G217B_predicted.fasta) and gene structures (G217B_predicted.gff3) of the GSC predictions are mirrored at http://histo.ucsf.edu/downloads/. Click here for file

Additional file 2 **Table S2**. CSV formatted table giving GSC predicted gene names corresponding to *H. capsulatum *G217B genes referenced in the text. As noted in the results section, the predicted gene structures are not necessarily identical to experimentally characterized transcripts. Click here for file

Additional file 3 **Table S3**. GFF3 formatted (tab delimited) table of detected TAR genomic coordinates. Coordinates are given relative to the 11/30/2004 GSC G217B assembly, which is mirrored at http://histo.ucsf.edu/downloads/F_HCG217B.fasta.041130.gz. Click here for file

Additional file 4 **Data S4**. WIG formatted plus strand tiling probe intensities mapped to the 11/30/2004 GSC G217B assembly, suitable for viewing in Gbrowse2 http://gmod.org/wiki/GBrowse. Click here for file

Additional file 5 **Data S5**. WIG formatted minus strand tiling probe intensities mapped to the 11/30/2004 GSC G217B assembly, suitable for viewing in Gbrowse2 http://gmod.org/wiki/GBrowse. Click here for file

Additional file 6 **Data S6**. FASTA formatted DNA sequences for the 264 novel TARs. Coordinates relative to the 11/30/2004 GSC G217B assembly are given in the FASTA header lines. Click here for file
